# Ultrafast Dynamics of Organic Exciton–Polaritons

**DOI:** 10.1002/nap2.70049

**Published:** 2026-03-20

**Authors:** Soham Mukherjee, David C. Bain, Gloria Davidova, Andrew Musser

**Affiliations:** ^1^ Department of Chemistry & Chemical Biology Cornell University Ithaca New York USA

**Keywords:** exciton polariton, polariton photochemistry, strong light‐matter coupling, transient absorption spectroscopy

## Abstract

Exciton–polaritons are increasingly proposed as a route to control molecular excited‐state dynamics through hybridization with light, with potential implications for nanophotonic and quantum photochemical applications. However, directly characterizing the dynamics and excited‐state signatures of exciton–polaritons remains a major challenge. In ultrafast transient absorption experiments, photoexcitation of polariton states typically results in a host of overlapping spectral responses that overwhelm any signals from the polariton population, leading to conflicting accounts of the photophysical pathways under strong coupling. In this study, we provide a framework to systematically identify the signatures of photoexcited polaritons through simultaneous control of optical cavity structure and excitation conditions. Under phase‐matched excitation, we directly track coherent polariton relaxation dynamics on sub‐100 fs time scales, revealing an ultrafast decay pathway that circumvents the intracavity dark states. We establish that the critical design parameter to achieve purely polaritonic dynamics is the degree of spectral overlap between the lower polariton and the tail of the molecular absorption. Our results provide a route to engineer coherent light–matter interactions at the nanoscale and call into question the prevailing framework of polariton photophysics, based on entropy‐driven scattering into intracavity dark states.

## Graphical abstract

This work reveals the conditions required to capture the coherent dynamics of organic exciton–polaritons. The ultrafast relaxation of these collective hybrid states is uniquely observed only when they sit beyond the tail of the molecular absorption. In this condition, they do not enter into equilibrium with intracavity dark states, raising questions about the prevailing model of polariton dynamics.

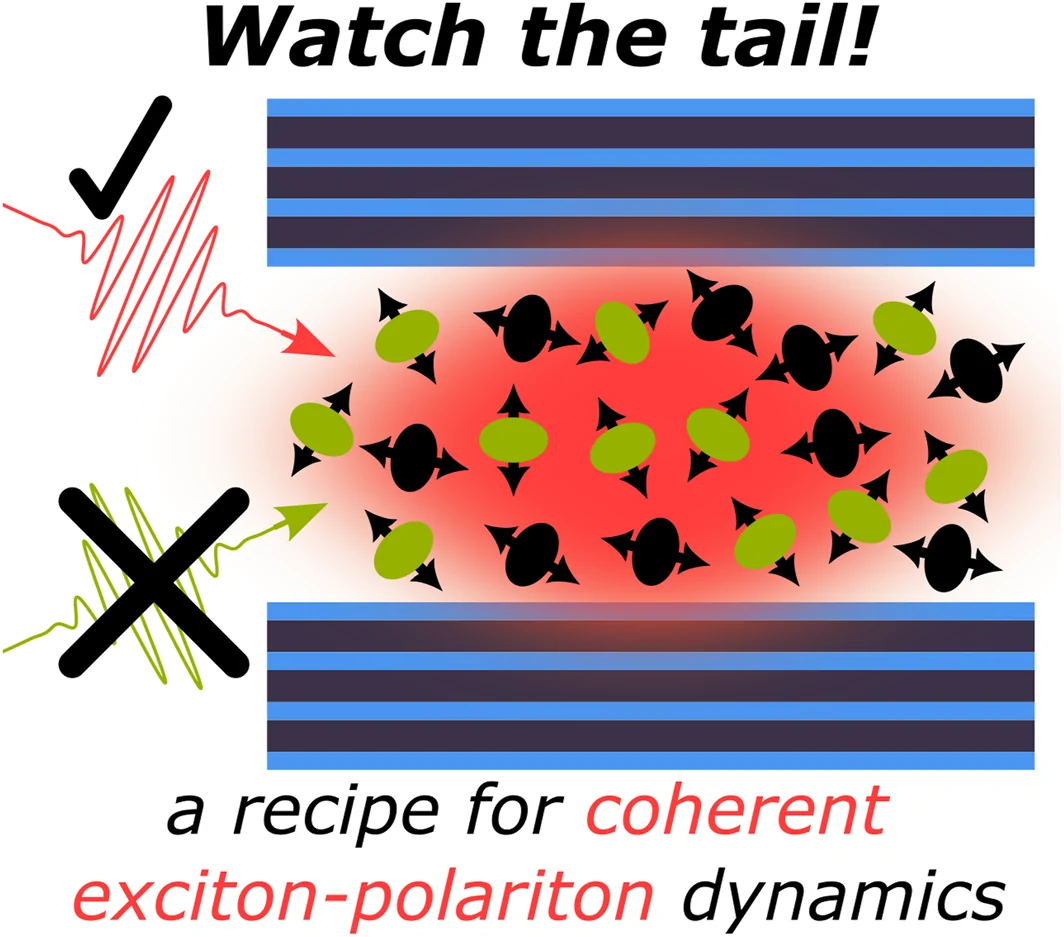

## 1 Introduction

Recent years have witnessed a surge of interest at the interface of photophysics and cavity quantum electrodynamics, particularly in exploring whether and how strong light–matter coupling can redirect excited‐state dynamics in organic semiconductors [1, 2, 3, 4]. The study of organic photophysics has principally centered around the behavior of excitons and charge‐separated species in isolated or aggregated molecules, but the incorporation of these systems into optical microcavities introduces new hybridized states exciton–polaritons, that may significantly perturb this landscape [3, 4, 5]. Exciton–polaritons arise when excitons interact strongly with a confined photonic mode, such as those present in microcavities or plasmonic resonators. This interaction is collective across all resonant excitons in the mode volume, leading to the formation of extensively delocalized new hybrid states. The resulting upper polariton (UP) and lower polariton (LP) are separated in energy by the Rabi splitting ℏΩ, which parameterizes the collective light–matter coupling strength, and they exhibit properties—for example, energy, effective mass, lifetime, nonlinearity—that arise from the weighted mixture of exitonic and photonic components [5, 6, 7, 8]. The balance between these components is readily tuned through the energetic detuning between bare photon and exciton states, the angle of incidence, and the density of absorbing molecules in the cavity, which maps onto ℏΩ. All told, strong coupling results in the formation of highly tunable coherent eigenstates that span the cavity volume. Their formation has been reported to alter molecular properties/processes such as charge and energy transport [9, 10, 11, 12, 13, 14, 15, 16], bulk photoisomerization kinetics [17, 18, 19], lasing thresholds [20, 21], and delayed emission from spin‐forbidden states [22, 23, 24, 25]. When formed by coupling to infrared vibrational absorption bands, polariton formation has been associated with surprising changes to the rate and selectivity of thermally driven chemical reactions [26, 27]. Though the mechanisms of these effects are not well understood [28, 29], they have inspired interest in using exciton–polaritons to redirect photochemistry, exploiting both the energetic tunability and the variable photonic versus matter character of the polariton wavefunction to reshape molecular potential energy surfaces [2, 29, 30, 31, 32, 33].

Such effects have yet to be conclusively observed, but they offer the tantalizing prospect of rationally enhancing the rates of processes such as singlet fission [34, 35], intersystem crossing [25, 36, 37], or photochemical charge transfer [38, 39, 40, 41]. Accordingly, there has been growing interest in probing excited‐state dynamics within the strong‐coupling regime, principally using transient absorption (TA) spectroscopy [41, 42, 43, 44, 45, 46, 47, 48, 49, 50, 51, 52, 53, 54, 55, 56, 57]. Results are most frequently interpreted in terms of the Tavis–Cummings model, which describes the collective coupling of N degenerate excitons with a single photonic mode. This framework predicts the formation of two bright polariton states UP and LP, separated by the collective ℏΩ as well as a pool of N−1 degenerate intracavity dark states (DS). Given the collective coupling regime is typically considered to involve vast numbers of molecules (of order 10^9^ or greater) [58, 59, 60], the DS dominate the density of states and thus cavity photophysics [5]. Extensive effort has thus been put into identifying the various artifacts that arise during TA measurements of Fabry–Perot cavities, from thermalization effects to Rabi contraction [48, 50, 51, 53, 61, 62]. The latter effect captures the transient reduction in ℏΩ due to the removal of molecules from the ground state by photoexcitation of LP, UP, or DS, and it can be particularly misleading as it causes a strong TA response at the LP and UP resonance energies that tracks with the exciton dynamics. Effectively, these efforts have centered on identifying what features are not the spectral signatures of photoexcited polaritons. As highlighted in recent theoretical work [63], it has proved much more challenging to determine what features actually are their spectral signatures.

The aim to capture the spectra and dynamics of photoexcited molecular polariton states, and to distinguish them from the signatures of cavity‐related artifacts or DS effects, poses stringent technical constraints. The constraints can be best appreciated within the simple Tavis–Cummings framework. In this scheme, the polariton wavefunction at any in‐plane wavevector can be decomposed into photonic and excitonic components, each weighted by its Hopfield coefficient $\gamma_{ex}$ or $\gamma_{ph}$. This picture leads to the prediction that the exciton‐polariton decay rate is governed by the corresponding weighted average of excitonic ($k_{ex}$) and photonic ($k_{ph}$) decay rates as $k_{pol}=\gamma_{ex}k_{ex}+\gamma_{ph}k_{ph}$ [5, 64]. Unless there is unexpected new physics that arises due to light–matter hybridization—for instance due to the dilution of photonic character across the ensemble in the presence of disorder [65, 66, 67]—we can consider this expression to represent a lower limit for the polariton decay rate as it does not factor in other potential population loss channels, such as scattering or decoherence, into the intracavity DS [68, 69]. The critical challenge, then, is that outside of trivial edge cases ($\gamma_{ph}\to 0$), $k_{pol}$ will be dominated by $k_{ph}$. In organic exciton–polariton systems, which are most frequently studied in Fabry–Perot cavities, $k_{ph}$ typically ranges from ${\left(∼10\;fs\right)}^{-1}$ in metallic structures to ${\left(100-300\;fs\right)}^{-1}$ in the high‐Q DBR cavities used for polariton condensation. Observing dynamics in this regime requires correspondingly short excitation pulses. This spectroscopic standard has, to our knowledge, only been met in the study of organic exciton‐polaritons once in measurements on Fabry–Perot cavities in 2011 [42] and again recently using Bloch surface wave polaritons where $k_{ph}$ is markedly longer [41].

Here, we lay out an experimental strategy to robustly capture the spectroscopic fingerprints and dynamics of excited LP states. We evaluate a series of moderate‐Q DBR cavities containing layers of Rhodamine 6G in either the weak‐ or strong‐coupling regime and with a range of active‐layer thicknesses (detuning) that scans the photonic mode across the Rhodamine absorption spectrum. Through careful comparison of the TA spectra and dynamics following direct excitation of the intracavity DS versus resonant pumping of the LP as appropriate with temporally compressed pulses, we identify the spectral and dynamical hallmarks of the coherent LP population. Notably, in almost all circumstances, the TA signal is dominated by the effects of DS population, regardless of sample structure or excitation scheme, and these take essentially the same form within the weak‐coupling regime. Only when the LP is tuned well beyond the intracavity absorption edge do we capture the coherent LP response, and in that case, it decays without entering equilibrium with the DS. These findings contradict the standard picture of exciton–polariton dynamics, and they highlight the underappreciated importance of the molecular absorption tails to understand organic cavity photophysics.

## 2 Materials and Methods

The active layer of our Fabry–Perot cavities is composed of Rhodamine 6G (R6G, Sigma Aldrich, Figure 1a), a xanthene‐based laser dye widely employed in photonic and optoelectronic applications due to its high fluorescence quantum yield, strong molar absorptivity, and reasonable photostability. We deposited pure films of R6G onto quartz‐coated glass substrates (Ossila) or sputter‐deposited DBRs using a thermal evaporator (Angstrom Instruments, 160°C; at a rate of 1 Å/s). Where necessary, we deposited nonabsorbing spacer layers of Tris (8‐hydroxyquinoline)aluminum(III) (Alq3, Sigma Aldrich) in the same system in sequential fashion (280°C; at a rate of 1 Å/s). We fabricated the bottom DBR mirrors through magnetron sputter deposition of alternating high‐ and low‐refractive index dielectrics TiO_2_ (at a rate of 0.3 Å/s) and SiO_2_ (at a rate of 0.4 Å/s) onto cleaned quartz‐coated glass substrates. Layer thicknesses were optimized to center the high‐reflectivity stopband near the R6G absorption peak (60 nm TiO_2_ and 100 nm SiO_2_; Figure 1b). Top DBR mirrors were deposited in the same system, directly on top of the R6G or Alq3 layer. We optimized the sample structure to compromise between enhanced polariton lifetime (small $k_{ph}$, requiring maximized reflectance) and signal strength in TA spectroscopy (requiring moderate light transmission through the cavity)—the cavities reported here used 3.5 pairs of TiO_2_:SiO_2_ layers for the bottom mirror and the top mirror, yielding a Q‐factor of 250 and an estimated $k_{ph}={\left(75\;fs\right)}^{-1}$.

**FIGURE 1:**
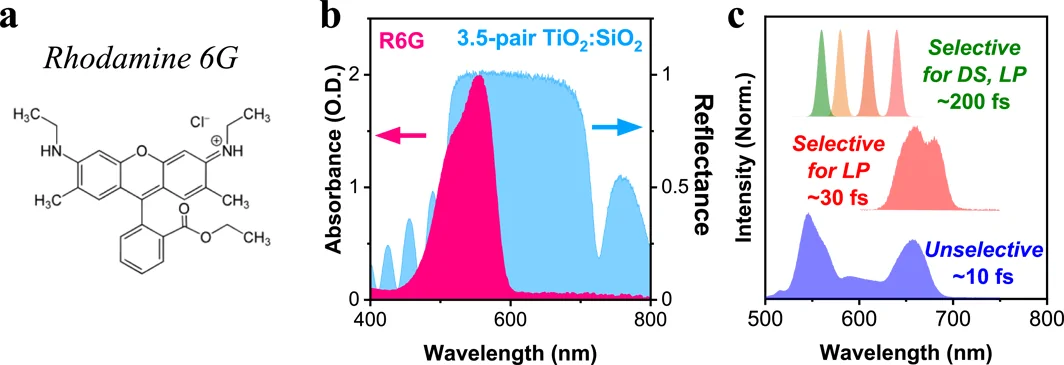
Materials and methods. (a) Molecular structure of Rhodamine 6G (R6G). (b) Absorption spectrum of thermally evaporated R6G film with thickness of 160 nm (pink) and reflectance spectrum at normal incidence of a 3.5‐pair bottom DBR mirror used in cavity fabrication (blue). Layer thicknesses are TiO_2_: 60 nm and SiO_2_: 100 nm. (c) Excitation pulse spectra used in TA measurements, providing control over state selectivity and temporal resolution.

We measured thin‐film UV–vis absorption spectra on a home‐built absorption spectrometer using a Xe plasma white‐light source (LDLS, Hamamatsu) and Avantes AvaSpec‐Mini4096 detector. To measure the reflectance of DBRs and characterize the angle‐resolved reflectance of our Fabry–Perot cavities, we incorporated this source and detector into a home‐built fiber‐coupled goniometer with two arms rotating between angles of incidence of 9° and 70°, with 0° defined as normal incidence.

We used two different TA spectroscopy setups to measure the excited‐state dynamics of our thin films and cavities, both operating in transmission geometry (Figure 1c). Following the method of Michail et al., [52] we ensured that both pump and probe had similar angle of incidence on the cavity, in practice no more than 8° apart. (1) Narrowband (∼10 nm FWHM) TA was performed using a commercially available Ultrafast Systems HELIOS spectrometer, driven by a Pharos‐10 W femtosecond pulsed laser (1030 nm, 180 fs, Light Conversion) operating at 8 kHz. Pump pulses were generated using an Orpheus optical parametric amplifier (Light Conversion) and tuned to specific transitions for state‐selective excitation. The resulting pulses had a duration of ∼200 fs, offering limited temporal resolution. (2) Ultrafast broadband TA was performed using a home‐built TA spectrometer driven by the same Pharos laser as previously reported [70]. We directed 6 W of the Pharos output into a HIRO harmonic module (light conversion) for frequency doubling (515 nm and 0.5 W) and subsequent sum frequency generation (343 nm and 0.375 W). The 343 nm output was used as the pump source for a noncollinear optical parametric amplifiers (NOPA). 10 mW of the Pharos fundamental was separately focused into a 3 mm sapphire window for white light continuum (WLC) generation to be used as a seed for the NOPA. The center of each WLC was selected by using a hard aperture, and it was subsequently passed through a 950 nm short‐pass filter before being focused onto a nonlinear crystal for amplification by its respective pump. The WLC was focused by detuning a spherical mirror used as the collection optic after the sapphire window. The pump and seed were temporally and spatially overlapped in a 2 mm BBO crystal (Eksma, *θ* = 31.5°, and Type I). After amplification, the NOPA output (typically ∼520–680 nm) was directed through a chopper (Thorlabs, MC2000B) and compressed using a combination of chirped mirrors (Laser Quantum, DCM9, and 20 bounces) and silica wedge pairs (Newport, 23RQ12‐02‐M). The full bandwidth pulse was typically compressed to ∼10 fs as confirmed by second harmonic generation frequency resolved optical gating (Figure S1). To enable selective excitation of the polaritons without fully sacrificing the temporal resolution, we also performed measurements with a modified NOPA with reduced bandwidth (∼625–700 nm) and a compressed duration of 30 fs (Figure S2). The pump pulses were then directed to a home‐built TA spectrometer, using WLC generated in a 3 mm sapphire window as the probe. After transmission through the sample, the probe was dispersed onto a CMOS detector (Stresing Entwicklungsburo, 3010, and HA:S12198‐1024). A step size of 5 fs was used during TA scans, and 4000 pump on/off spectra were collected per time point in shot‐to‐shot fashion using custom LabVIEW software.

## 3 Results

### 3.1 Cavity Coupling and Detuning Series

In Figure 1b, we present the steady‐state absorption spectrum of a 160 nm evaporated film of R6G. The spectrum exhibits a pronounced absorption maximum at 560 nm with a shoulder at 520 nm. This band primarily corresponds to the 0–0 and 0–1 vibronic peaks of the S_0_→S_1_ electronic transition, but the intensity of the 0–1 peak is enhanced relative to dilute solution (Figure S3). This behavior is characteristic of H‐type dimer and larger aggregate formation in R6G, and in principle, it leads to complex excited‐state relaxation dynamics due to energy migration through the heterogenous ensemble of monomeric and aggregated states [71, 72, 73]. However, these processes occur on longer timescales than the polariton lifetime of interest here (∼75fs). Moreover, we maintain the same active‐layer composition for all samples, so it is possible to control for these effects while maintaining a simple sample structure. The exceptional oscillator strength of this film relative to the moderate absorption linewidth (∼350 meV FWHM, considering the 0–0 and 0–1 peaks together) suggest it should be suitable for strong light–matter coupling. In the same panel, we plot the reflectance spectrum of a bottom DBR used for cavity fabrication, which reveals that the stopband is well overlapped with the full molecular absorption.

In order to distinguish between the signatures of photoexcited polaritons and other cavity‐related effects, we prepared three different types of DBR cavity: empty, weakly coupled, and strongly coupled (Figure 2a,c,e). All cavities were designed with a separation between mirrors of 160–220 nm, tuned to place one of the photonic modes in or near resonance with the R6G absorption maximum. In empty cavities, the active layer consisted of a pure thermally evaporated film of Alq3, which has negligible absorption the spectral range of interest for R6G (450–650 nm and see Figure S4). As shown in Figure 2b, the structures sample photonic mode minima of 580 nm (EC‐580), 610 nm (EC‐610), and 635 nm (EC‐635). For weakly coupled cavities, we incorporated a thin pure film of R6G (20 nm), on top of a thick Alq3 spacer (140 nm) to shift it away from the cavity field antinode and minimize light–matter coupling strength. The R6G film was capped by a further layer of Alq3 (10–30 nm) to shift the mode position. The angle‐resolved reflectance in Figure 2d shows photon mode minima of 585 nm (WC‐585), 605 nm (WC‐605), and 625 nm (WC‐625); in all cases, the mode crosses directly through the R6G absorption band without noticeable change in dispersion, confirming that these samples are in the weak‐coupling regime. To achieve strong light–matter coupling, the entire cavity volume is filled with a film of R6G (160–190 nm). In these structures, angle‐resolved reflectance reveals clear splitting of the photonic mode as it passes through resonance with the R6G absorption band and evident leveling off of the lower‐energy mode as it approaches the R6G absorption edge at high angle (Figure 2f). These features are hallmarks of polariton formation, with a splitting of ∼0.6 eV between UP and LP that places the system firmly in the strong‐coupling regime. Across the detuning series, the LP minimum spans 585 nm (SC‐585), 615 nm (SC‐615), and 640 nm (SC‐640). In the thickest cavity SC‐640, a new faint mode can be detected immediately to the red of the exciton transition (arrow in Figure 2f, right)—this feature corresponds to the LP corresponding to the next‐order photonic mode (arrows in Figure 2b,d), which can interact with R6G due to the extremely strong absorption of the pure film. The presence of this secondary LP does not affect our analysis below.

**FIGURE 2:**
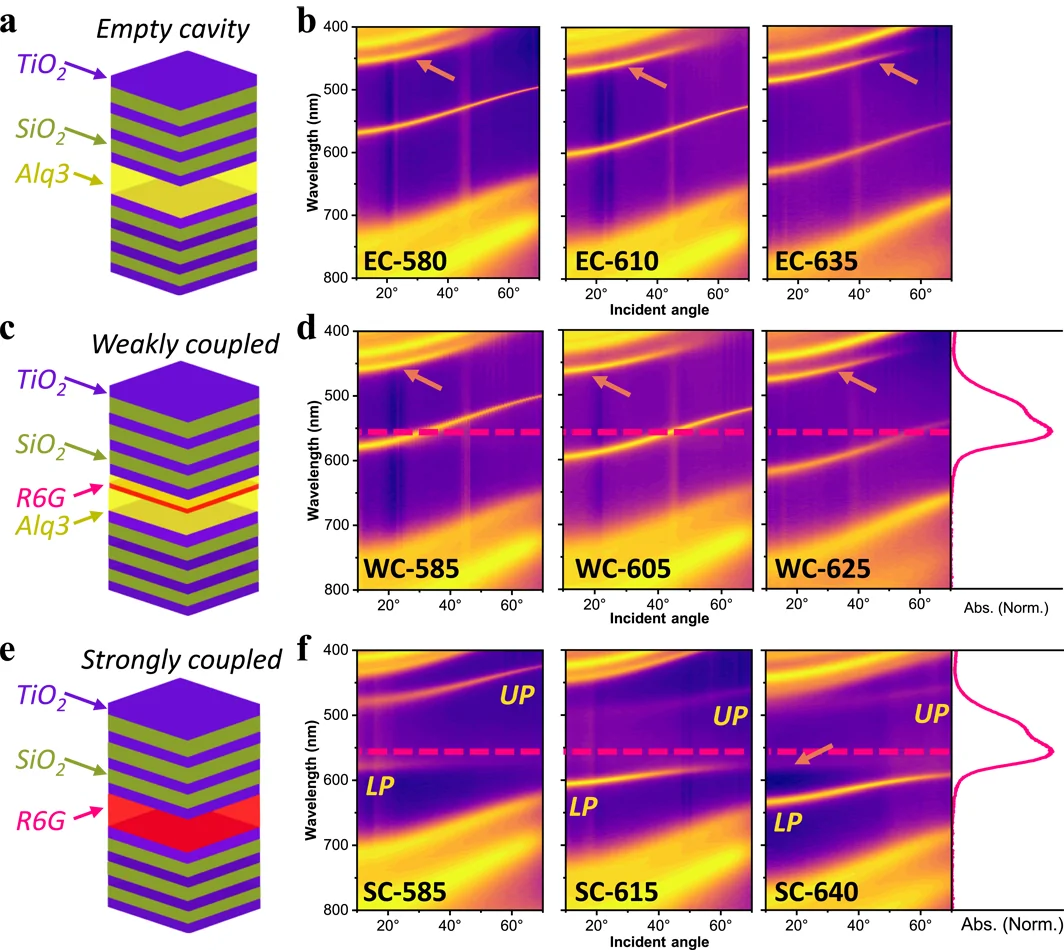
Cavity coupling and detuning series. (a) Schematic structure of empty cavity containing only Alq3, which does not absorb in this spectral range. (b) Angle‐resolved reflectance of empty cavities with progressively thicker Alq3 layer from left to right. The orange arrow indicates a higher‐order photonic mode that enters the DBR stop‐band. (c) Schematic structure of weakly‐coupled cavities containing a thin off‐center R6G layer between two Alq3 spacers. (d) Angle‐resolved reflectance of weakly‐coupled cavities with progressively thicker top Alq3 layer from left to right. The orange arrow indicates a higher‐order photonic mode that enters the DBR stop‐band. The dashed pink line highlights the R6G absorption peak (normalized spectra on right). (e) Schematic structure of strong‐coupled cavities containing a pure R6G layer. (f) Angle‐resolved reflectance of strong‐coupled cavities with progressively thicker R6G film from left to right. The orange arrow indicates a secondary LP that arises from the higher‐order photonic mode. The dashed pink line highlights the R6G absorption peak (normalized spectra on right).

### 3.2 Excitonic Versus Photonic Response: Weak Coupling

We studied all cavities containing R6G with TA spectroscopy under two basic excitation conditions. We used ‘non‐resonant’ excitation at 560 nm to directly photoexcite the exciton reservoir or intracavity DS (which are not fully dark in the presence of disorder [65, 67, 74]). We distinguish this condition from ‘resonant’ excitation, where the pump is tuned to the energy of the photonic or LP mode. For these experiments, we determine the mode energy to pump directly in the TA spectrometer through observation of the transmission peak, typically using an angle of probe incidence (relative to normal incidence) of ∼6°. Empty cavities were measured only under resonant excitation, presented in Figure S5. In the WC series, nonresonant excitation yields the same basic behavior regardless of detuning: a derivative‐like lineshape centered at the wavelength of the optical mode (Figure 3b). This feature decays over more than 100 ps, well in excess of the sub‐ps cavity photon lifetime. Over this timescale, we observe subtle spectral evolution best captured by the gradual blue‐shift of the zero‐crossing between ΔT/T<0 and ΔT/T>0 regions. As shown below, these dynamics match with those of the bare (cavity‐free) film and the spectral evolution we capture in Figure 3b reflects excitonic spectral dynamics within the R6G film. This behavior is not surprising—in the absence of strong coupling or light–matter hybridization, there should be no reason to anticipate significant changes to the intracavity exciton dynamics in the cavity. Yet, we emphasize that the derivative‐like lineshape observed here matches the feature most often observed and discussed in the context of polariton spectroscopy [43, 44, 45, 46, 47, 48, 49, 50, 51, 52, 53, 54, 55], despite the complete absence of polaritons. It evidently need not be related to polariton physics. Indeed, we can readily interpret these spectra as a simple optical effect. Photoexcitation of R6G results in bleaching of its strong ground‐state absorption, slightly reducing the intracavity refractive index throughout the R6G absorption band. The photon mode is consequently blue‐shifted, leading to enhanced transmission (ΔT/T>0) at shorter wavelengths and increased reflectance (ΔT/T<0) at the original mode position [61, 62].

**FIGURE 3:**
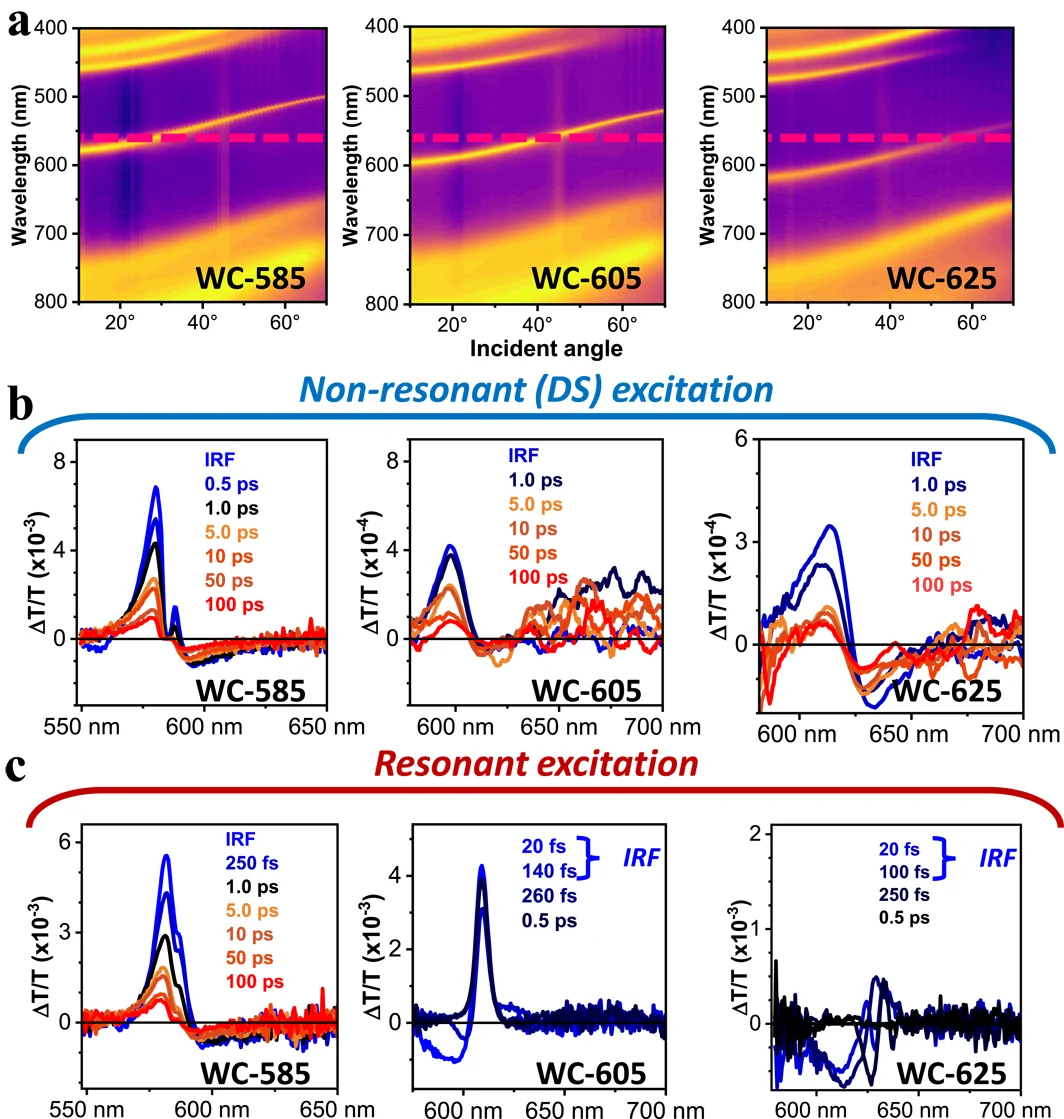
R6G dynamics under weak coupling. (a) Angle‐resolved reflectance data of the weakly coupled cavity series, duplicated from Figure 2d. The R6G absorption peak is indicated with the dashed line. (b) TA spectra of the WC series following nonresonant narrowband excitation at 560 nm. (c) TA spectra of the WC series following resonant narrowband excitation at the wavelength of the photonic mode. The ΔT/T>0 signal that persists beyond 0.5 ps in WC‐605 and WC‐625 is pump scatter. IRF denotes signals within the 200‐fs instrument‐response function of the narrowband TA experiment.

We find that resonant narrowband excitation of the photonic mode makes no difference in the case of WC‐585, presumably because the mode overlaps with substantial R6G absorption, leading to similar excitation of long‐lived R6G exciton states to the case above (Figure 3c, left). However, for the redder photonic modes WC‐605 and WC‐625 (Figure 3c, middle and right), we observe starkly different behavior under resonant narrowband pumping. Though the spectra are marred by strong pump scatter, the response is evidently characterized by a derivative‐like feature of opposite sign to the case of nonresonant excitation. Moreover, it is extremely short‐lived, with instrument‐limited decay that is complete (aside from pump scatter) within 500 fs. These spectra are presented on larger scale in Figure S6. This decay is in reasonable agreement with the expected sub‐ps photonic lifetime of these cavities. This behavior is nearly identical to the characteristic response of empty cavities in Figure S5. We interpret this behavior as the characteristic response of a purely photonic excitation, which causes a red‐shift of the mode in the presence of intracavity photons.

### 3.3 Excitonic Versus Photonic Response: Strong Coupling

We observe the same basic behavior for nonresonant excitation of strong‐coupled samples SC‐585, SC‐615, and SC‐640. The TA response is dominated by a derivative‐like feature centered near the ground‐state LP minimum (gray shading in Figure 4b,c). Because the UP states fall in a spectral range where the white‐light supercontinuum probe is very weak, and the UP additionally exhibits minimal transmission, we are unable to capture any TA response in the UP region where subtle differences in the polaritonic TA response have been predicted [63]. Within this constraint, these results for the SC series are effectively equivalent to those for the WC series, including the lineshape of the optical response, the gradual blue‐shift of the zero‐crossing from ΔT/T<0 to ΔT/T>0, and the persistence of strong signal to beyond 100 ps. The similarity to WC signals and the lifetime ∼1000‐fold longer than expected for exciton–polaritons in our cavities suggest that even though this sample supports polaritons, the transient optical response under nonresonant excitation does not reflect population in the LP state. Instead, these features can be readily explained in terms of effects such as Rabi contraction—in which reduction of the ground‐state population due to photoexcitation leads to transient reduction in ℏΩ—and the mode‐shifting refractive index effect discussed for the WC series. We thus interpret this type of optical response as purely indicative of intracavity DS dynamics [48, 50, 51, 53, 61, 62].

**FIGURE 4:**
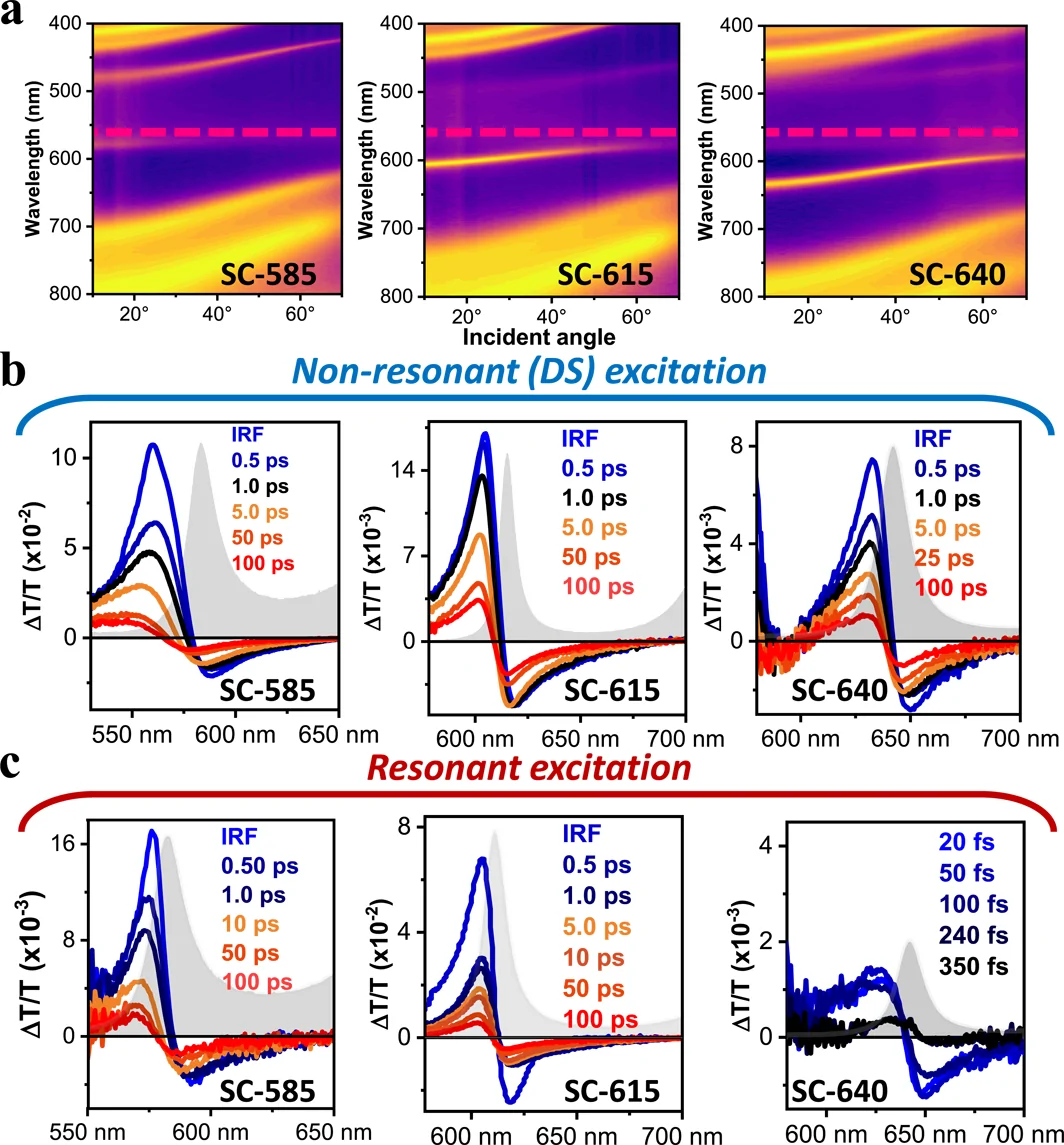
R6G dynamics under strong coupling. (a) Angle‐resolved reflectance data of the strong‐coupled cavity series, duplicated from Figure 2f. The R6G absorption peak is indicated with the dashed line. (b) TA spectra of the SC series following nonresonant narrowband excitation at 560 nm. The gray shaded spectra are cuts of the steady‐state reflectivity at the angle of incidence of the probe, taken at the location of the TA measurement. (c) TA spectra of the SC series following resonant excitation of the LP. SC‐585 and SC‐615 were measured using resonant narrowband excitation at 585 and 615 nm, whereas SC‐640 was measured using the 30‐fs resonant selective broadband pulse shown in Figure 1c. The gray shaded spectra are cuts of the steady‐state reflectivity at the angle of incidence of the probe, taken at the location of the TA measurement. Slight differences in steady‐state reflectivity between b and c are due to the measurement being performed at a different location on the same sample. IRF denotes signals within the 200‐fs instrument‐response function of the narrowband TA experiment.

Resonant narrowband excitation of SC‐585 leads to no discernible change in the TA signal (Figure 4c, left); we capture the same spectral shape, dynamics, and gradual blue‐shift. Here, too, the optical response is characteristic of intracavity DS: resonant excitation on its own is an insufficient condition to capture true polaritonic effects, especially where the LP strongly overlaps with the absorption tail. However, we begin to detect distinct effects in resonantly excited SC‐615 (using selective narrowband pump; Figure 4c, middle). The basic derivative lineshape of the signal persists, but on sub‐200‐fs timescales (IRF, blue), this feature is notably sharper, with much less of the tail that extends beyond 630 nm in the nonresonant case. Importantly, this new spectral profile rapidly (< 1 ps) evolves into the same broader derivative feature previously observed, which then decays over the usual ∼ 100 ps range. This behavior points to the involvement of two distinct states or processes—tentatively, fast LP decay followed by slow excitonic/DS dynamics—but they are challenging to disentangle and dominated by the excitonic behavior. Finally, in the most red‐detuned cavity SC‐640, resonant excitation results in a unique optical response. We again observe a derivative‐like feature, but it is more evenly balanced between ΔT/T<0 and ΔT/T>0 than under any other conditions, and it fully decays with a ∼75‐fs lifetime. Over the course of this decay, the zero‐crossing does not dynamically blue‐shift as appears to be typical of DS dynamics, pointing to a different electronic origin for the signal. Although this behavior is similar to the purely photonic responses in resonantly‐pumped WC‐625 and the empty cavities, the sign of the derivative is flipped—the signal shape instead matches the characteristic feature of the other experiments in which excitonic states are populated. That is, the response for resonantly excited SC‐640 exhibits properties of both the purely photonic signals (∼ 75 fs lifetime, limited by cavity *Q*‐factor) and excitonic signals (a derivative signal that arises from a transient blue‐shift of the photon/LP mode). It qualitatively resembles the signature of photoexcited surface‐bound exciton‐polaritons reported previously [52]; hence, we assign the signal here to photoexcited LP population. Only under this last condition is the contribution from intracavity DS eliminated.

### 3.4 Summary of Cavity Optical Response

We note that the spectral differences between coherent LP states (Figure 4c, right) and exciton‐induced changes to the photonic or LP mode (Figures 3b and 4b,c left and middle) are subtle. The photoexcited LP signal shows no dynamic blueshift of the zero‐crossing and the derivative lineshape is more symmetric, but only through careful comparison between multiple detunings and excitation conditions can this distinct response be recognized. From these experiments, we determine the minimal conditions required to observe the true polariton response: (1) cavity photon/LP lifetime longer than the pump pulse duration [61], (2) resonant excitation at the correct energy and angle of incidence [52], (3) and sufficient detuning that the LP does not overlap with the tail of the exciton absorption. The latter point is critical and often overlooked, but it explains the essential difference between resonant excitation of SC‐585, SC‐615, and SC‐640. We return to this issue below. First, we summarize the two spectroscopic regimes captured in our experiments.

When the pump overlaps spectrally with any portion of the intracavity exciton absorption, the spectral response is the same regardless of weak versus strong coupling. It manifests as a derivative‐like lineshape due to a transient blueshift of the cavity mode (purely photonic or LP). This feature decays with dynamics that track the intracavity exciton dynamics, and there is no discernible difference between weak‐ and strong‐coupling regimes (Figure 5, blue). This comparison suggests that any changes to exciton photophysics in strong‐coupled cavities following nonresonant excitation are simply due to the cavity environment [62] rather than a consequence of polariton formation. In our observations, the only way to achieve a notably different spectroscopic regime is by exciting resonantly with the photonic/LP mode in a region where there is no spectral overlap with the exciton absorption (even the tail). This regime gives rise to a similar derivative‐like lineshape, but it does not shift over the course of the decay. Only here do we detect a significant difference between the weak‐ and strong‐coupling regimes—the ΔT/T feature takes an opposite sign under weak coupling—but even so the dynamics are effectively equivalent (Figure 5, red) and governed by the cavity Q‐factor. In these conditions, we see no evidence for involvement of the intracavity DS, whether through direct excitation or scattering from the LP.

**FIGURE 5:**
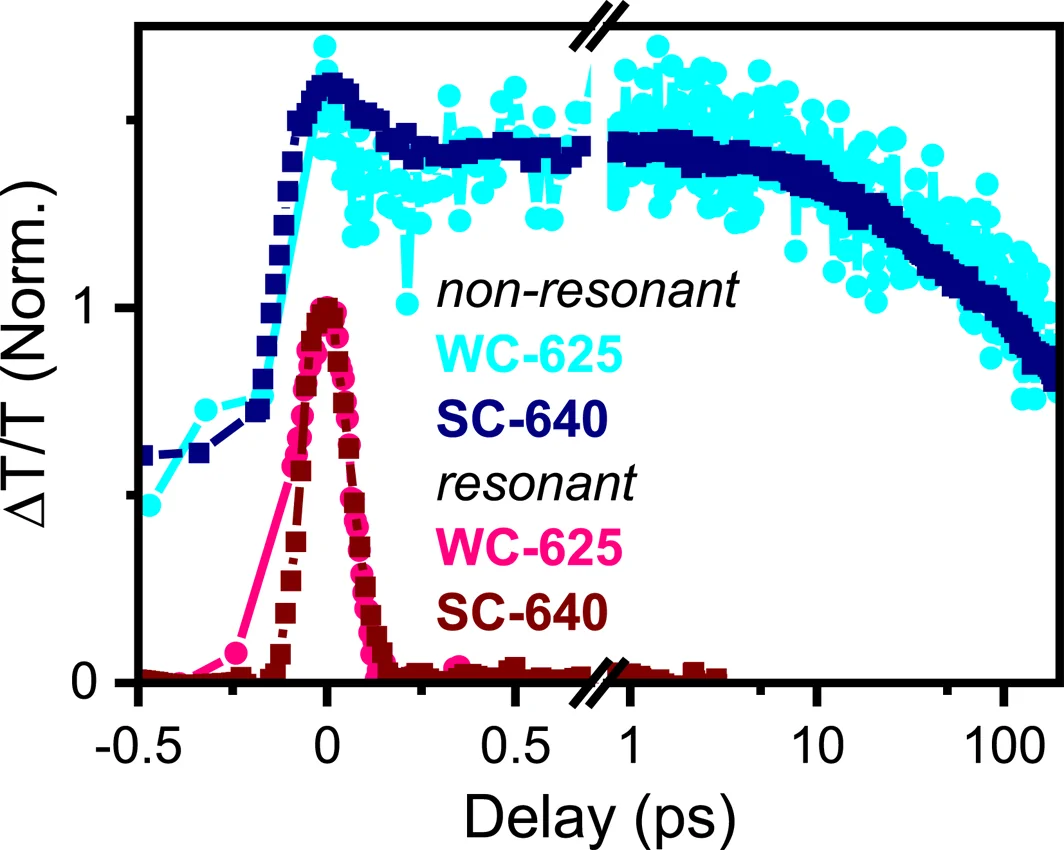
Kinetics under different coupling regimes. Comparison of the integrated TA kinetics at the photoinduced absorption (ΔT/T<0) feature of the derivative‐like lineshape of WC‐625 and SC‐640, following nonresonant narrowband excitation of the DS at 560 nm versus resonant narrowband excitation at 625 nm or resonant selective broadband excitation 640 nm.

### 3.5 Matching Film and Cavity Dynamics

Given the primary optical response in most experimental conditions is dominated by intracavity exciton dynamics, an important question is whether the cavity environment changes them. Indeed, there have been reports of both enhanced and suppressed exciton–exciton annihilation in R6G cavities, attributed to changes in the range of energy transfer [55, 75]. In other materials, strong coupling has been reported to result in changes to bulk photophysical pathways due to alterations to the potential energy surfaces or altered thermodynamic ordering of states in the cavity [14, 25, 44, 45, 46, 49, 76]. To probe such effects, we performed TA measurements on bare films of R6G, excited at the absorption peak at 560 nm. The TA spectra in Figure 6a clearly exhibit a change in lineshape over the course of the population decay: a fast decay in the ΔT/T peak at 560 nm and the stimulated emission (ΔT/T>0) band extending up to 725 nm, followed by a persistent (> 100 ps) ground‐state bleaching band shifted toward shorter wavelengths. This behavior is typical of high‐concentration R6G films, and it reflects the evolution of a heterogeneous population of monomeric and aggregate excitations [71, 72, 73]. The fast decay at 560 nm and in the stimulated emission can be attributed to monomeric sites, which are effectively quenched in bulk films and are susceptible to singlet‐singlet annihilation. The longer decay at 520 nm is reported to reflect the dynamics of weakly emissive aggregates. In Figure 6b, we plot the kinetics extracted for the strong‐coupled cavities excited at 560 nm alongside various kinetic traces taken from the bare‐film data. If we compare the cavity kinetics to the thin‐film data taken at the same probe wavelengths (same‐color symbols vs. lines in Figure 6b), there appears to be a systematic enhancement in lifetime under strong coupling. However, the probe at the redder wavelengths where the LP is observed predominantly samples the short‐lived monomeric states. If we instead compare the cavity dynamics to the 520 nm bleaching peak, which captures the evolution of both monomeric and aggregated states, we obtain excellent agreement to the longer‐time dynamics. This comparison reveals that the cavity signal—which derives from polariton contraction due to depopulation of the R6G ground state—reflects the full excited‐state population evolution, not just the dynamics of the monomeric states closest in energy. It thus combines the responses of short‐lived monomeric and long‐lived aggregated species, with the balance dependent on the LP spectral position. Once we correctly understand the origin of the strong‐coupled cavity signal, then, we find that polariton formation has no significant impact on the photophysics of the R6G film under nonresonant excitation. The intracavity exciton dynamics are minimally perturbed inside and outside the cavity, from weak to strong coupling, for a wide range of detunings. We find that this null result applies over a range of laser powers. Across our accessible range, we find no difference in the power dependence—and thus the annihilation behavior—of R6G films and cavities (Figure S7).

**FIGURE 6:**
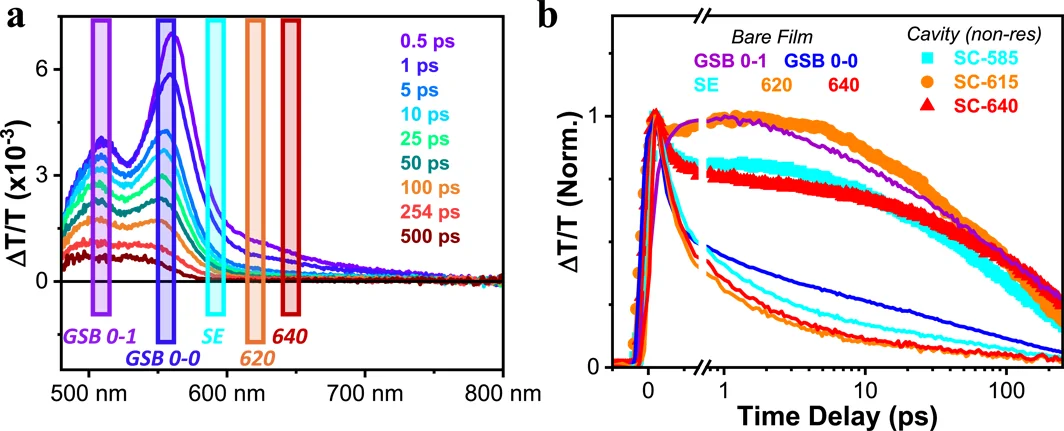
Bare film versus cavity dynamics. (a) TA spectra of R6G thin film photoexcited with narrowband excitation at 560 nm. Colored bars indicate integration regions analyzed for kinetics. (b) TA kinetics of R6G thin film (lines) versus the photoinduced absorption (ΔT/T<0) feature of the derivative‐like lineshape of the SC series, following nonresonant excitation at 560 nm. Comparison at equivalent probe wavelengths (same color) suggests a significant increase in lifetime within the cavity, but all cavity kinetics simply reflect a combination of GSB 0–1 (aggregate) and GSB 0–0 (monomeric) behavior.

## 4 Discussion

Our experiments provide a basic framework to capture the spectral signatures and dynamics of photoexcited exciton–polaritons and to distinguish them from trivial cavity‐related effects [50, 51, 61, 62, 77]. Numerous reports have expended significant efforts to anticipate and describe these cavity‐related features through optical modeling, chiefly using transfer‐matrix methods [48, 49, 53, 61, 62]. Although these approaches provide valuable insight into potential artifacts, they are limited as a tool for pinpointing the signatures of coherent polariton populations. Implicit in this approach is the idea that any features not captured in the ‘trivial’ optical model should be signatures of excited polariton states, reflecting their unique excited‐state manifold [48]. The validity of that assumption rests on the model taking into account all possible artifact contributions—Rabi contraction [62], intracavity excited‐state absorption [51], hot electron thermalization in the mirrors [50], thermal expansion [61, 78], and changes in sensitivity to anisotropy [79], to name a few—which poses a significant challenge. Our complementary, fully experimental approach shows that direct comparison to weakly coupled cavities, together with careful control of the excitation conditions, permits ready determination of the polaritonic response and the impact of intracavity DS. Building on the method of Michail et al., [52] we find that in Fabry–Perot cavities the excitation energy and angle of incidence are critical parameters to control. Moreover, polaritons only dominate the signal when the LP is sufficiently negatively detuned to exhibit minimal overlap with the tail of the molecular absorption and is selectively pumped with a carefully tuned excitation pulse. Indeed, when we excite cavity SC‐640 with a 10‐fs broadband pulse that spans LP and DS, the TA response is effectively indistinguishable from the (slow) non‐resonant response captured in Figure 4b (Figure S8). Within these constraints, it should be possible to directly observe the spectral structure of LP/UP states recently predicted by Philipp et al., [63] which would permit conclusive distinction between polaritonic and dark‐state signatures. Our measurements already point to the hybrid character of the LP state manifested in its TA response: a short lifetime governed by the rapid photonic decay rate $k_{ph}$, combined with a lineshape that resembles polariton contraction due to transient bleaching of R6G. However, we emphasize that this framework sets up a stringent condition for studying the spectral signatures and dynamics of photoexcited LP. The need for selective excitation requires that the LP be beyond the molecular absorption tail—indeed, even SC‐615 appears to barely permit observation of the LP response superimposed on the DS dynamics, despite being 55 nm red‐shifted from the R6G absorption peak. This condition is most readily met under significant negative detuning, meaning it would be particularly challenging to study the ultrafast dynamics for a relatively flat (e.g., positively detuned) dispersion where the LP‐DS energetic separation is small or for polaritons with a relatively high exciton fraction. It may be interesting to explore how this behavior changes as a function of Rabi splitting, especially toward the onset of ultrastrong coupling where the dispersion flattens regardless of detuning, though our results indicate that great care must always be taken about the absorption tail. A combination of the fully experimental method described here with optical modeling approaches [48, 49, 53, 61, 62] may help to deconvolve the ultrafast polariton dynamics from more complex mixed‐excitation cases such as resonantly‐pumped SC‐615.

Most of our results can be readily reconciled with the standard framework arising from the Tavis–Cummings model, in which the UP and LP coexist with a vast reservoir of intracavity DS. Many theoretical and experimental studies frame the dynamics in the cavity in terms of equilibrium between these populations [3, 4, 5, 53, 60]. In particular, the vastly larger density of states in the DS can result in an effective entropic driving force to maintain population there rather than the lower‐lying polaritons [69, 80]. One effective way to recast this balance is in terms of the free energy of the coherent LP/UP versus DS, in which case the DS are effectively stabilized relative to the LP, despite absorbing/emitting at higher energies [69]. The DS population should thus dominate the equilibrium with LP, regardless of which state is photoexcited. Consistent with this idea, numerous previous studies have found that the photophysical dynamics in strong‐coupled cavities inevitably follow the lifetime of the intracavity DS, even under resonant excitation of the LP, and the presence of the rapid decay pathway $k_{ph}$ scarcely impacts the evolution of the system [44, 45, 51, 52, 53, 81, 82, 83]. The scale of the corresponding entropic driving force can be estimated from the energy gap between the LP and the corresponding DS, typically taken at the exciton absorption maximum and neglecting the role of spectral bandwidth. In many reported systems, this energy gap amounts to hundreds of meV [46, 47, 53, 81, 84], a scale that can be easily accounted for by the vast number of molecules simultaneously coupled in the collective coupling regime [69].

This scheme can explain why, in almost every experimental configuration above, we only observe signatures of the DS population (through its impact on the cavity transmission spectrum). However, our result on resonantly excited SC‐640 calls this simple model of discrete states into question. The LP–DS energy spacing in SC‐640 can be estimated at 120–270 meV, with the lower value taken from the R6G absorption edge (605 nm) and the upper one from the peak at 560 nm. This spacing easily falls within the range of other assigned entropic effects, and we would anticipate that excitation of the LP should trigger evolution into the same LP‐DS equilibrium reported elsewhere. And yet, resonant excitation of the LP results in no population of the DS. This behavior is not unique—we find analogous effects in reported data on ultrafast polariton transport. In a variety of organic polariton systems, the coherent LP population can be identified by its ballistic transport, but in most instances, these LP states do not decay into the DS nor leave a trail of slow‐moving DS in their wake [10, 11, 12, 13]. Indeed, to our knowledge, there are no reports which (1) apply sufficient temporal resolution to capture the distinct LP state and (2) observe sequential relaxation of this LP into the DS, despite the centrality of this concept to the field. Instead, whenever the DS are observed they are present from the earliest observable timescale.

To understand this behavior and recast previous studies, we note the critical role of the absorption tail. In our measurements above, only when the LP was moved well beyond the low‐energy tail of the R6G absorption (SC‐640) could the unique LP response be detected. Where the LP overlaps with the tail (e.g., SC‐615), the data can be readily interpreted in terms of cavity‐ or LP‐enhanced absorption into excitonic tail states, with no need to invoke entropic effects. The apparent LP–DS equilibrium in our data is then due to direct excitation of the DS (which due to disorder are never rigorously dark) through the ‘filter’ of the LP mode. Indeed, we can obtain the same basic behavior of SC‐615—namely, a fast coherent LP decay superimposed on long‐lived DS dynamics—in cavity SC‐640 by tuning the angle of incidence to ∼30° so that the LP peak is located at ∼620 nm (Figure S9). The same picture applies to most previous studies of organic exciton–polariton dynamics, where the LP is typically very close to the exciton absorption, even when it is well‐separated from the peak [46, 47, 53, 68, 69, 81, 84]. Outside of edge cases such as SC‐640, then, we find that the spectroscopy of organic polaritons can be best understood in terms of cavity‐induced modification of the excitonic absorption and emission spectra, and it may not be helpful to analyze these systems in terms of unique polaritonic states. As for cases such as SC‐640, where a distinct decay pathway is achieved under resonant excitation, the results qualitatively resemble those obtained for purely photonic excitations (EC‐635 and WC‐625), but the Rabi‐contraction‐like transient blueshift indicates that hybridization with matter states has altered the spectroscopy. This dual behavior follows naturally from the density‐of‐states picture of strong coupling [65]. The matter density of states, and thus ‘matter’ properties, such as exciton/DS lifetimes, are scarcely impacted by strong coupling, since the polaritons constitute a negligible proportion of the total ensemble of states. On the other hand, the photonic density of states is completely altered by polariton formation, leading to new spectroscopic behavior. It remains an open question whether the matter character of the LP can uniquely impact its spectroscopy beyond simple Rabi contraction—for instance through singlet fission [35] or new nonlinear effects. This analysis suggests that the most promising route to harness polaritons for novel photochemical effects is to focus on photon‐mediated processes, such as transport, energy transfer, or delayed emission, rather than matter‐based electronic relaxation dynamics.

## Author Contributions

**Soham Mukherjee:** methodology, investigation, formal analysis, writing – original draft. **David C. Bain:** investigation. **Gloria Davidova:** investigation. **Andrew Musser:** conceptualization, funding acquisition, writing – review and editing, visualization, methodology, project administration, supervision.

## Conflicts of Interest

The authors declare no conflicts of interest.

## Supporting information


Supporting Information S1

## Data Availability

The online version of this paper includes Supplementary Information, including experimental details and figures. The datasets generated and/or analyzed during the current study are available from the corresponding author on reasonable request.
