# Supplementary material for: Ultrafast Dynamics of Organic Exciton–Polaritons
Source: Nanophotonics. 2026 Mar 20;15(6):e70049. doi: 10.1002/nap2.70049 (PMC13642613; doi:10.1002/nap2.70049)
Supplement: Supplementary file 1 [file NAP2-15-e70049-s001.pdf]

Supplementary Information

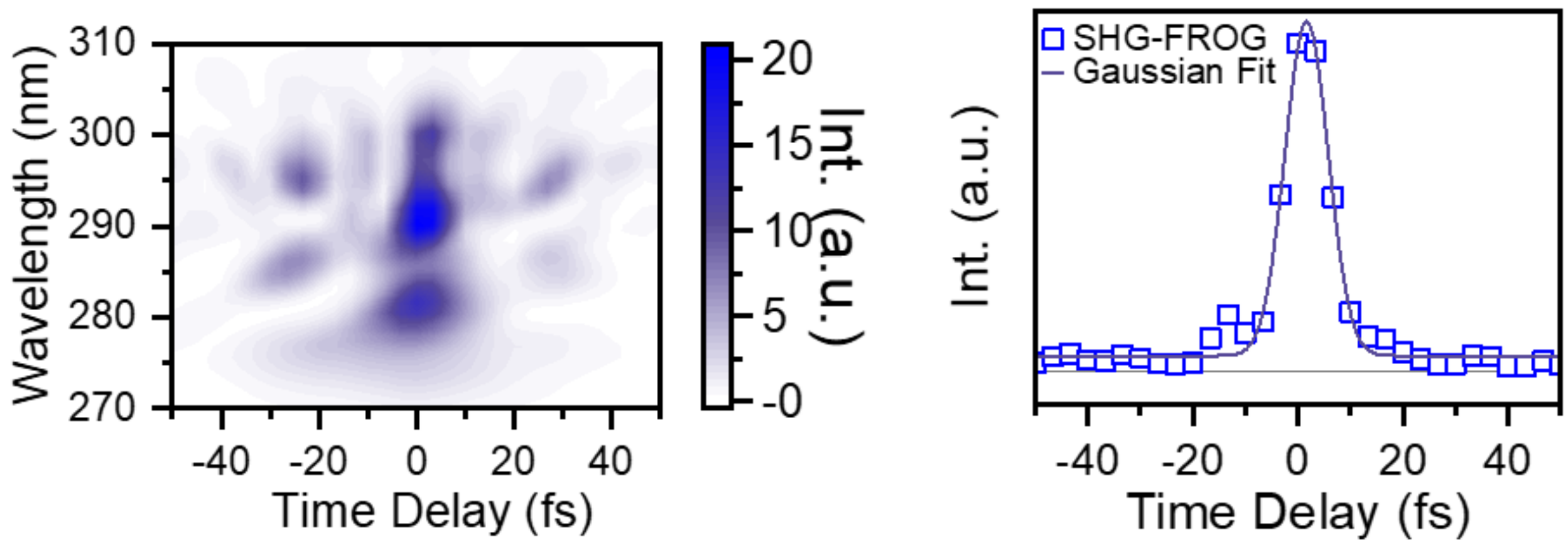

Figure S1: Pulse characterization for the unselective broadband excitation pulse used in ultrafast TA measurements. (a) SHG-FROG trace of the resonant excitation pulse and (b) Gaussian fit showing FWHM ~10fs.

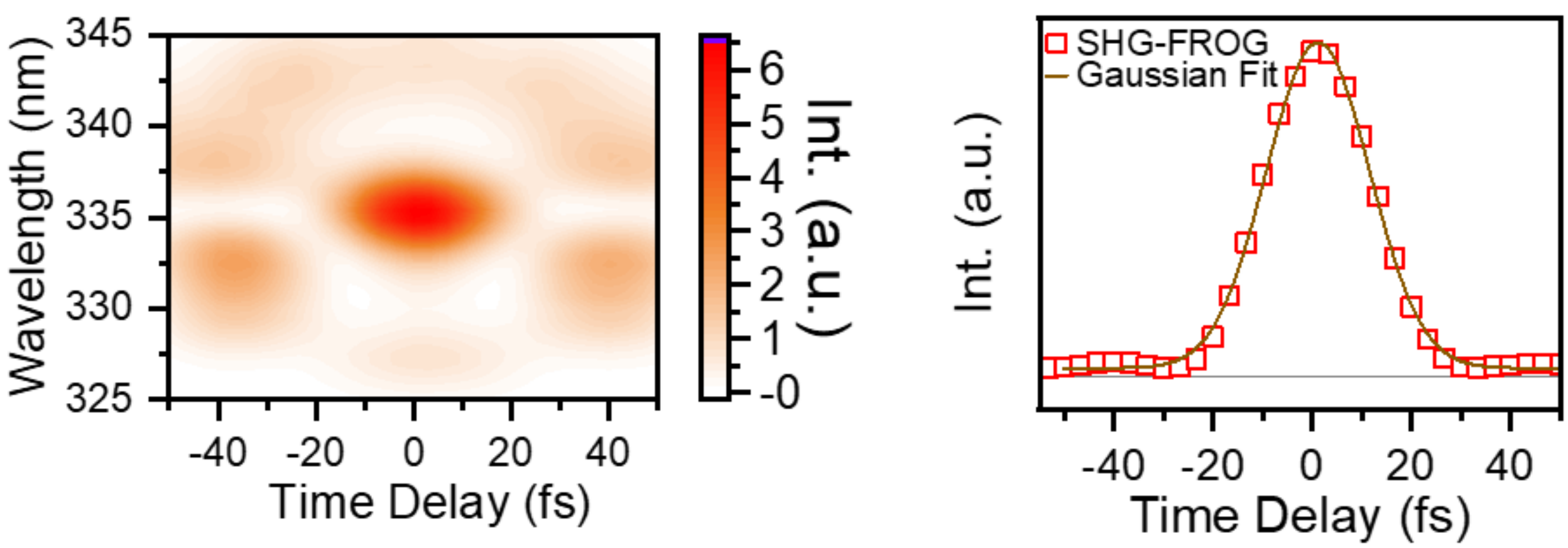

Figure S2: Pulse characterization for the selective broadband excitation pulse with resonant bandwidth (resonant to LP in corresponding cavity with no excitation towards the reservoir) used in ultrafast TA measurements. (a) SHG-FROG trace of the resonant excitation pulse and (b) Gaussian fit showing FWHM ~30fs.

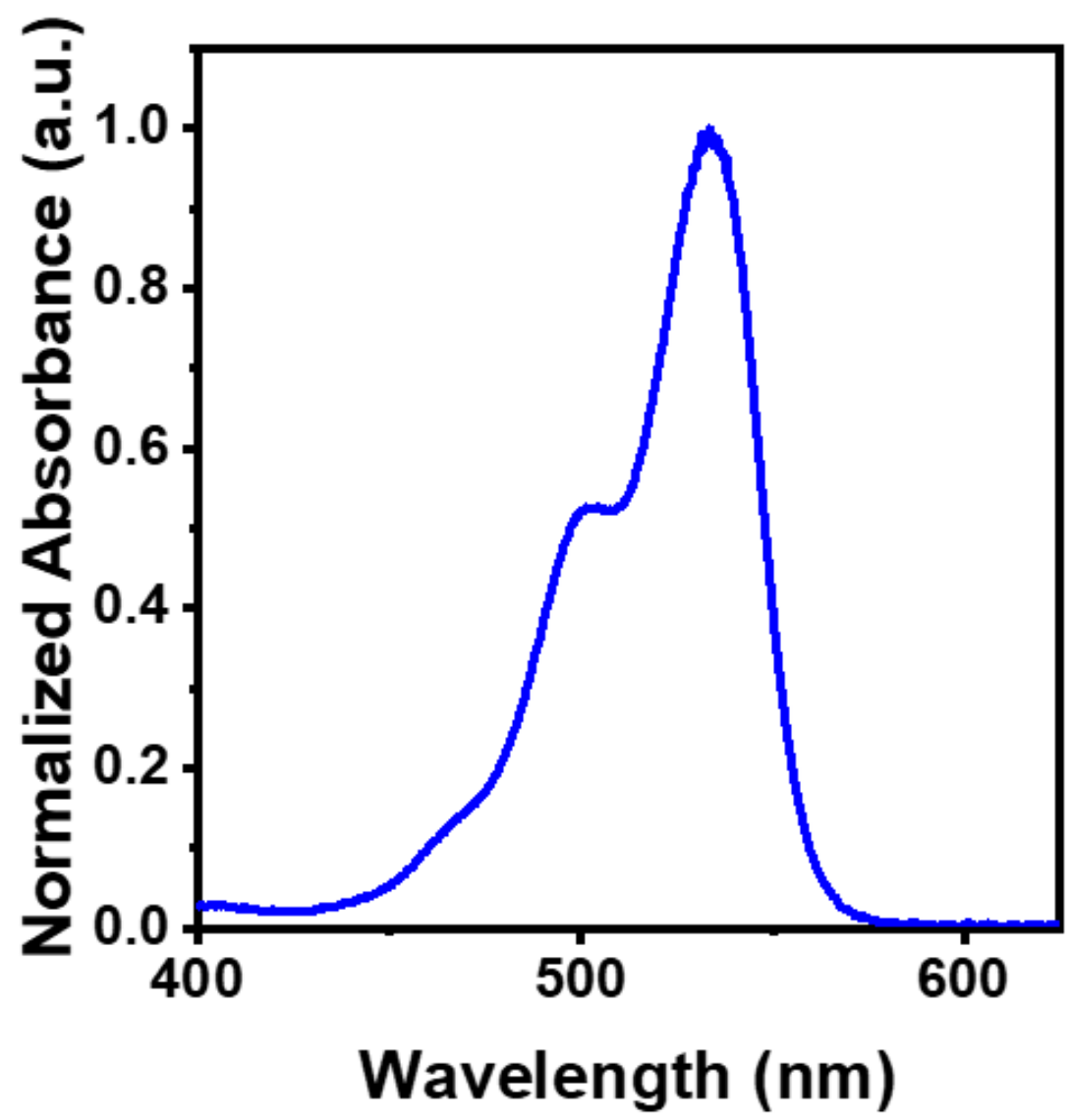

Figure S3: Absorption spectrum of a moderately dilute solution of R6G (0.5 mg/ml) in chloroform.

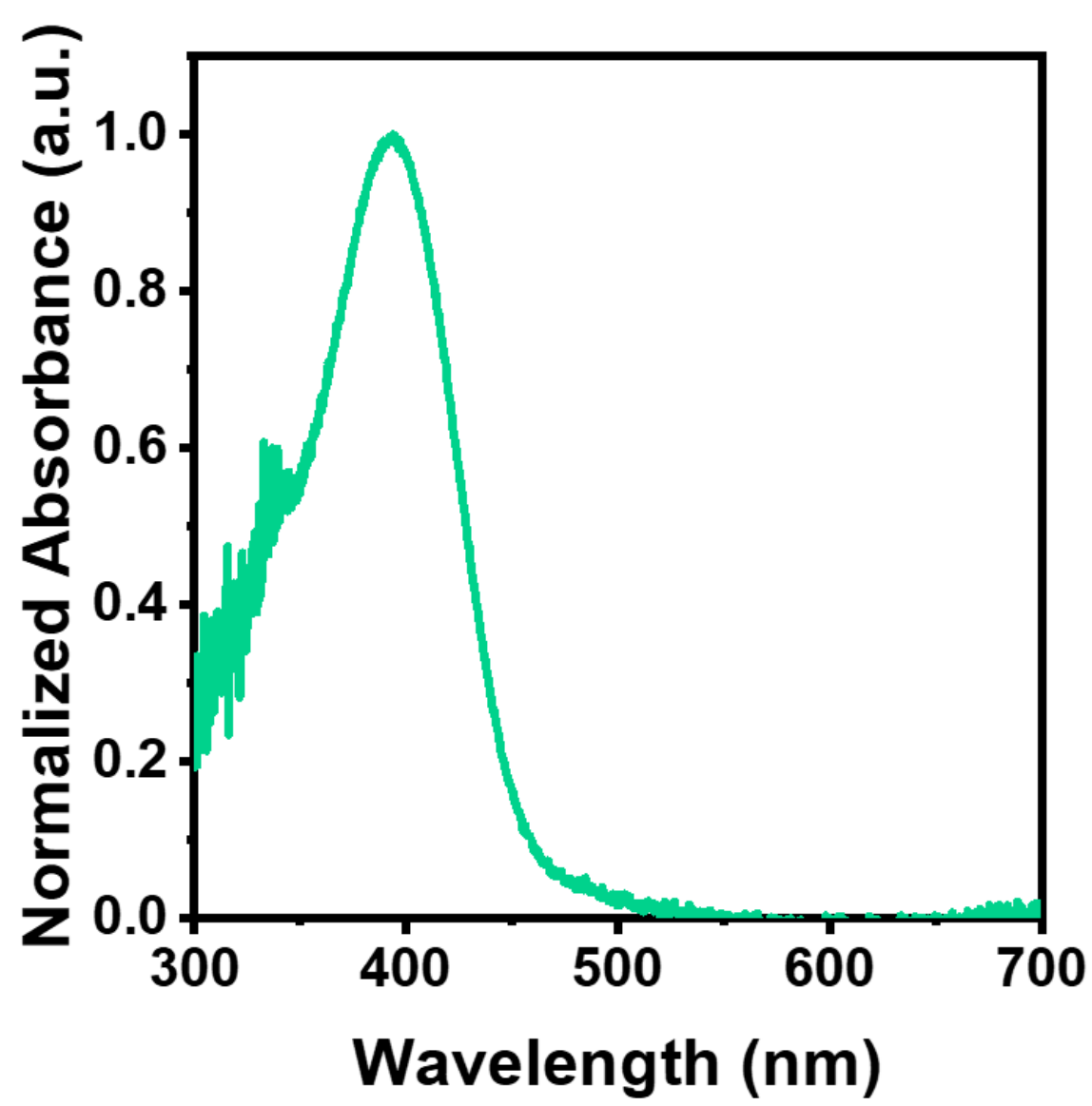

Figure S4: Absorption spectrum of an evaporated Alq3 thin film.

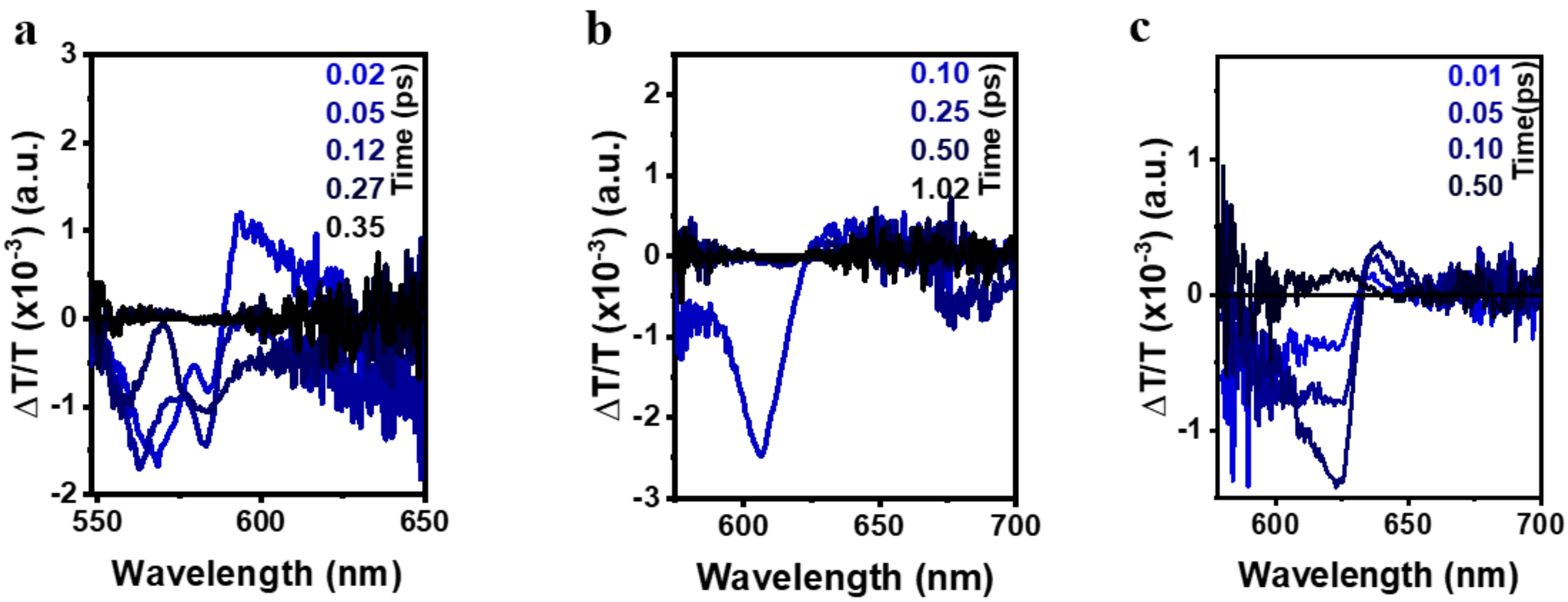

Figure S5: TA Spectra of Differently Detuned Empty (Alq3) cavity under resonant excitation condition: (a) EC-580; (b) EC-610; (c) EC-630.

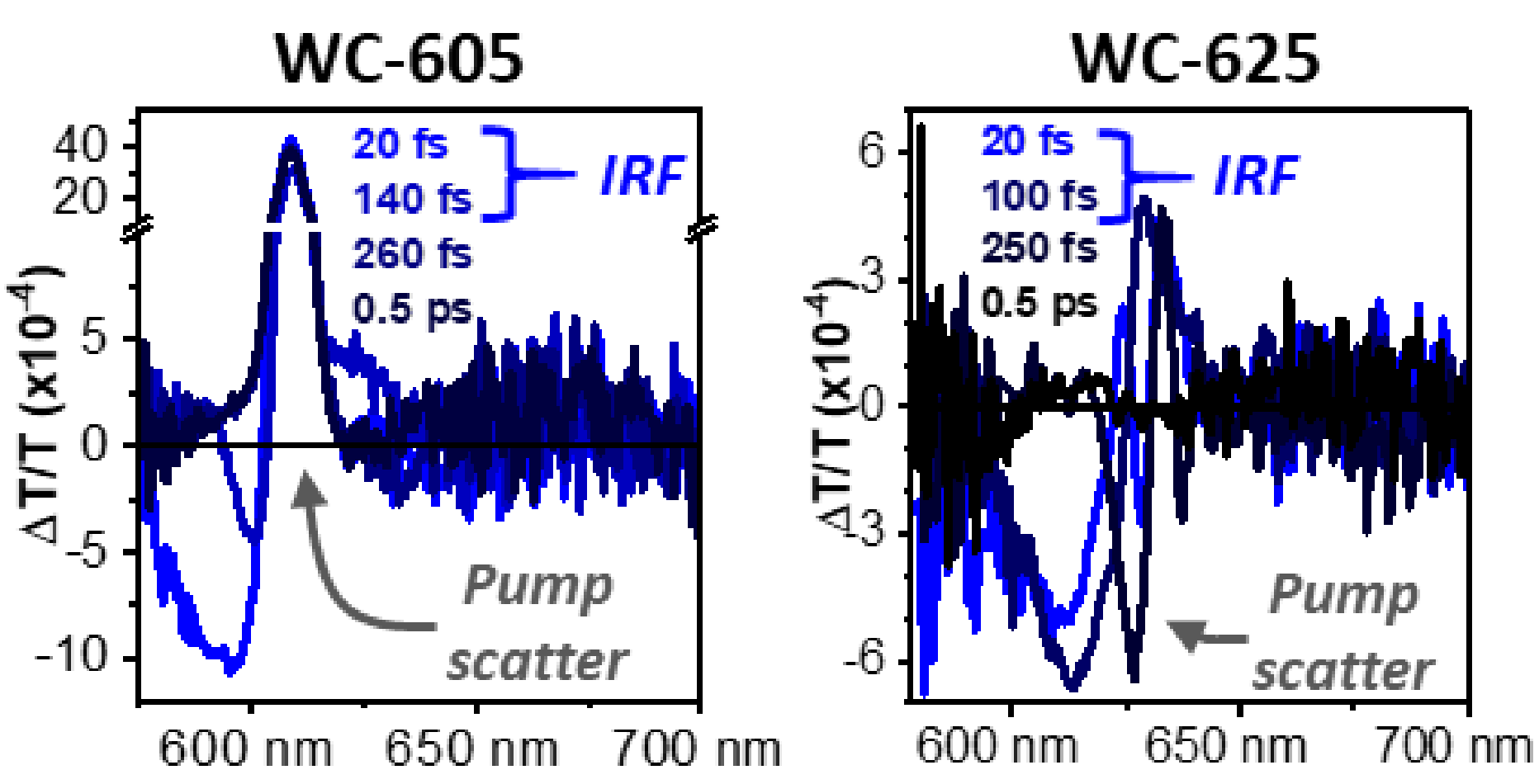

Figure S6: Zoomed-in view of weakly-coupled cavity data from Figure 3c to highlight the PIA evolution.

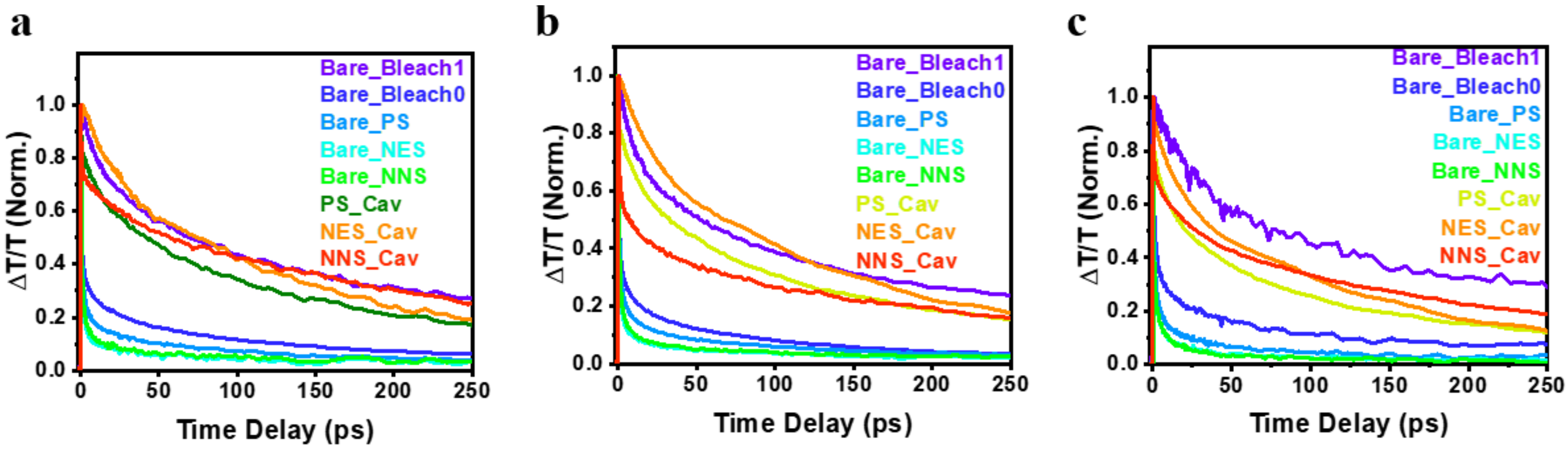

Figure S7: Comparison of Kinetics between R6G bare film and differently detuned strongly coupled cavities under different pump power of non-resonant reservoir excitation: (a) 50nJ; (b) 100nJ; (c) 250nJ.

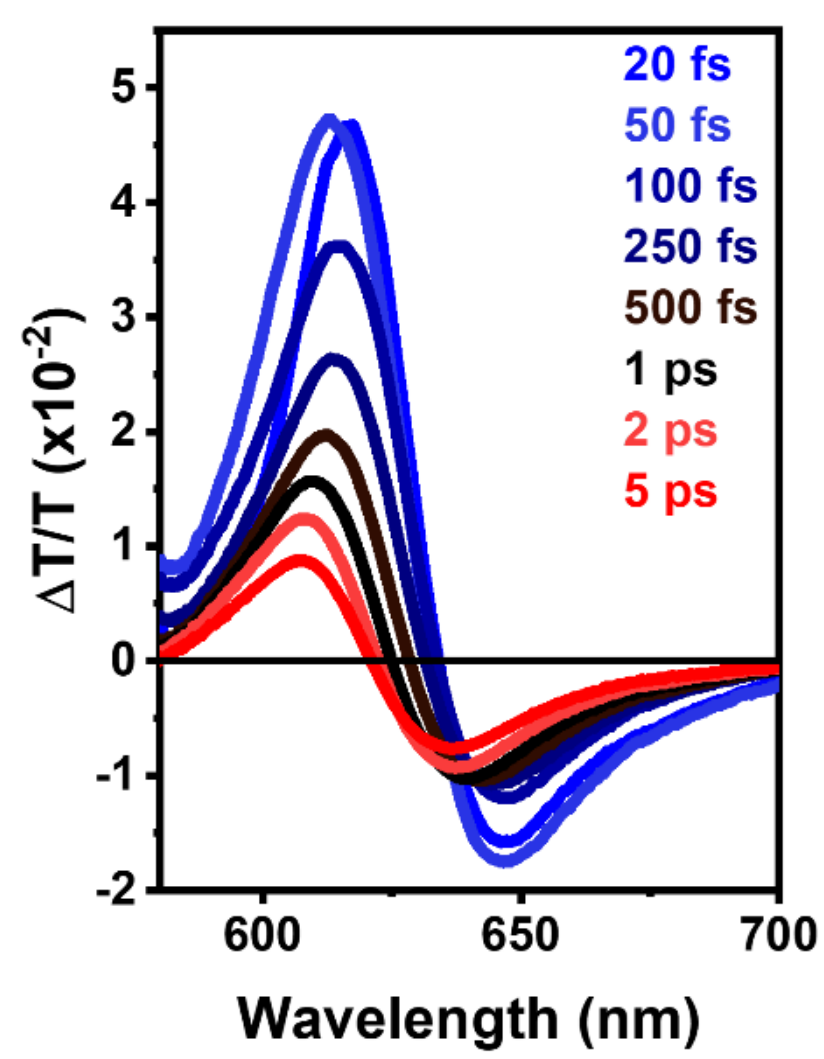

Figure S8: TA Spectra of negatively detuned strong-coupled cavity SC-640 under compressed broadband excitation with ~10fs pump duration. The signal is dominated by the long-lived DS response, similar to SC-615, and the DS signatures are equivalent to those observed under narrowband excitation.

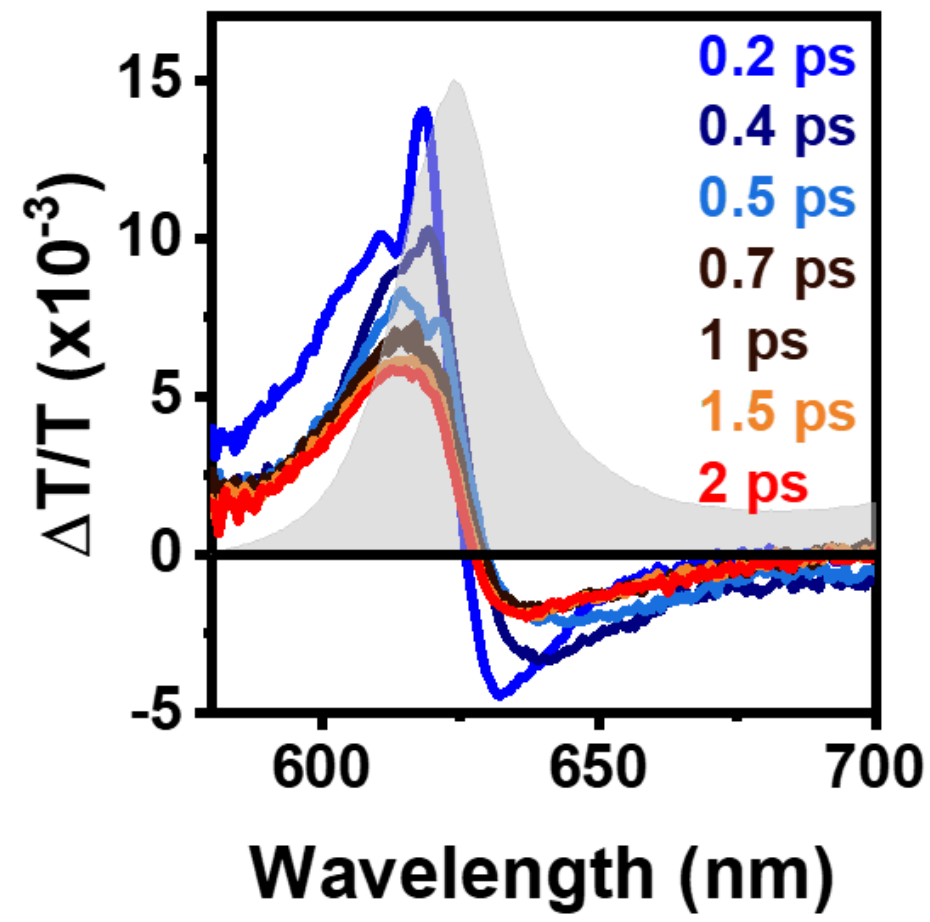

Figure S9: TA measurement of cavity SC-640 at a higher angle of incidence. The cavity was tilted so that the probe angle of incidence was ~30°, and the narrowband resonant pump was tuned to 620 nm. In this condition, the LP observed in probe transmission (grey spectrum) sits at ~620 nm, and the TA response closely resembles the mixed LP and DS response of resonantly excited SC-615 presented in the main text.

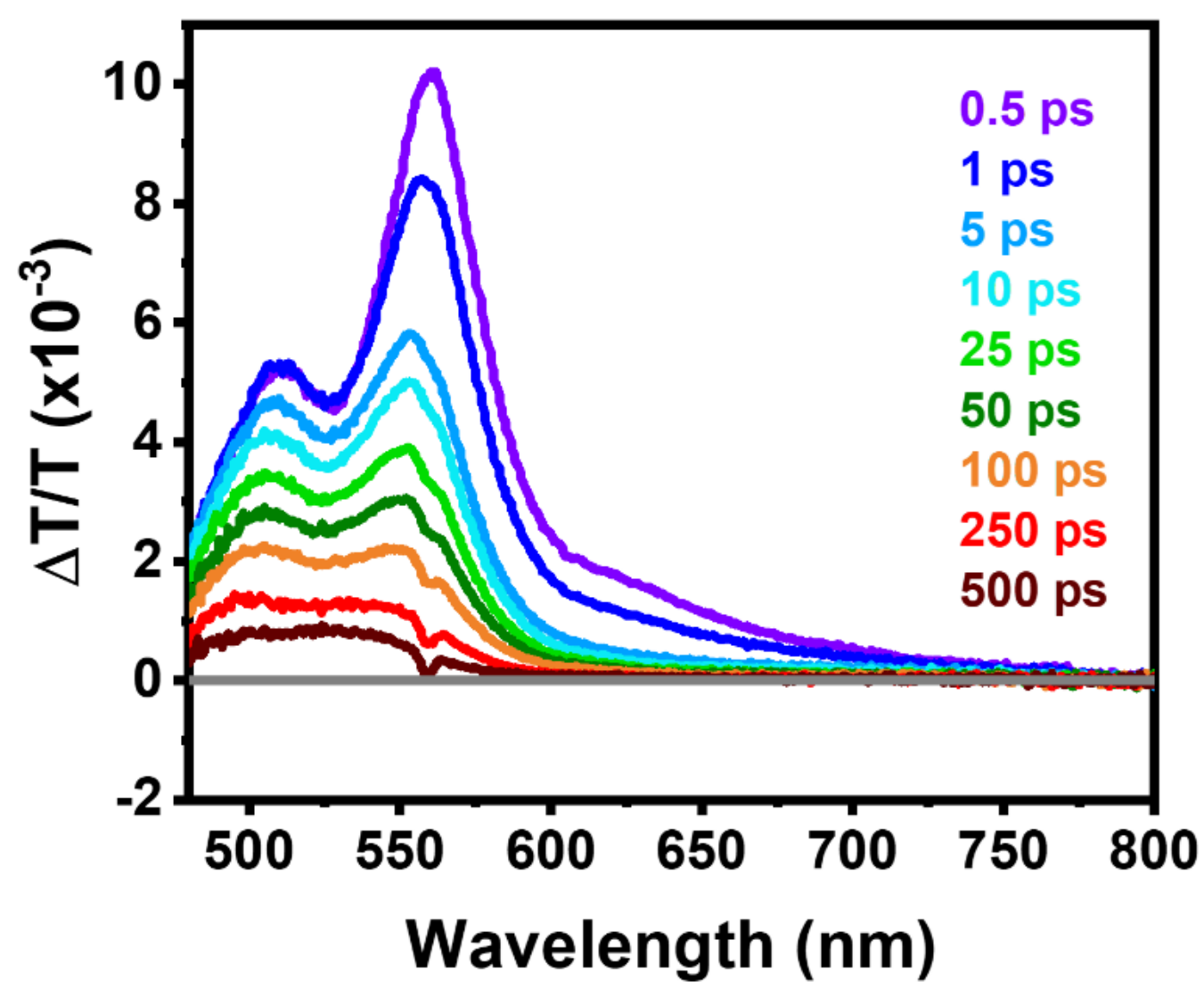

Figure S10: TA spectra of Alq3-R6G-Alq3 thin film photoexcited with narrowband excitation at 560 nm. The dynamics and spectral shape show no influence from the presence of R6G-Alq3 interfaces.
